# Tandem Mach Zehnder Directional Coupler Design and Simulation on Silicon Platform for Optical Coherence Tomography Applications

**DOI:** 10.3390/s20041054

**Published:** 2020-02-15

**Authors:** Yi-Ting Lu, Benedictus Yohanes Bagus Widhianto, Shih-Hsiang Hsu, Che-Chang Chang

**Affiliations:** 1 Department of Electronic Engineering, National Taiwan University of Science and Technology, Taipei 10607, Taiwan; M10702306@mail.ntust.edu.tw (Y.-T.L.); M10702821@mail.ntust.edu.tw (B.Y.B.W.); 2 Graduate Institute of Translational Medicine, College of Medical Science and Technology, Taipei Medical University, Taipei 11031, Taiwan

**Keywords:** Mach-Zehnder, directional coupler, optical coherence tomography

## Abstract

We design and compare the splitting ratio wavelength flatness of directional coupler (DC), Mach-Zehnder directional coupler (MZDC), and tandem MZDC. All coupler responses are analyzed, and tandem MZDC performance is the best in the wavelength insensitivity compared with the other two. An MZDC with any coupling ratio could be utilized to match the maximum flatness in a 40-nm wavelength range. To extend a broad flatness range, the tandem MZDC is proposed and still follows the Mach Zehnder structure taking two MZDCs as couplers connected through a decoupled region. Unlike DC, MZDC with the flat wavelength response has a non-linear output phase. Hence, using two wavelength-insensitive MZDCs as the coupling function in a tandem MZDC could demonstrate a more extensive decoupled phase term to maximize the flat wavelength response. The tandem MZDC theoretically demonstrates the splitting ratio with 100-nm flatness in the wavelength range from 1250 nm to 1350 nm. Finally, a point spread function through the tandem MZDC shows a 24-dB signal-to-noise ratio improvement in optical coherence tomography applications.

## 1. Introduction

Optical coherence tomography (OCT) is a high-speed imaging modality that is developed rapidly. OCT performs a high-resolution three-dimensional image by measuring the echoes of back-reflected and backscattered light [1]. OCT with medical and industrial applications can further be used quantitatively, such as for ophthalmology, dentistry, and dermatology, and qualitatively such as for angiography [2]. There are two common types of OCT existing, time-domain OCT and Fourier-domain OCT. Fourier-domain OCT owns sensitivity advantages and faster scanning time compared with time-domain OCT [3].

Silicon-on-insulator (SOI) has been developed as a common substrate for both optical and electronic applications. This technology also offers the potential as a critical platform for optoelectronic circuits. Hence, this kind of technology somehow can transform a bulky OCT-system into a compact silicon-photonics chip. Some OCT on-chip configurations have been made and published [4,5,6], and all of these works were made to obtain an OCT chip with a performance at least equal with traditional OCT. One of the crucial components in OCT on-chip is the optical power divider. It has an essential function as an interferometer, which is combining the reflection power from the reference and sample. In swept-source OCT, a power divider can also be applied as an auxiliary interferometer for data resampling to suppress nonlinearity [7]. The most common approach of producing optical power splitting is using a standard directional coupler (DC) because any arbitrary ratio can be achieved by the proper coupling length adjustment [8]. This simple design is susceptible to the operating wavelength. According to the coupled-mode theory, the coupling coefficient and propagation constant contribute to the amplitude and phase terms of the coupling power, which implies that the coupling performance would vary with the wavelength variation [9].

In the OCT system, we need a wavelength-insensitive coupler since the Fourier-domain OCT high-axial resolution depends on the broad wavelength response of guided wavelengths [1,2]. There have been several approaches to demonstrate the wavelength insensitivity through the optical coupler, such as multimode interference [10], asymmetry [11], adiabatic control [12], bending [13], and genetic process [14]. The propagation constant and coupling coefficient-related waveguide structures were manipulated to illustrate the above broad wavelength response. One of the solutions to obtain a compact, lower insertion loss [4] and flat wavelength-dependent coupler is using a Mach-Zehnder configuration over a directional coupler [15,16]. The Mach-Zehnder directional coupler (MZDC) could be constructed by two-directional couplers, connected through a short delay length in the uncoupled region, and demonstrate arbitrary optical splitting ratios with insensitivity to operating wavelengths compared with the traditional DC. The universal method to obtain parameters in MZDC configuration is already used widely. As far as we know, using this parameter, we can obtain a nearly 40-nm-flat bandwidth response with less than 1% difference in the splitting ratio.

For the OCT system-on-chip, previously, Culemann et al. laid out eight independent directional couplers-based interferometers in one single robust glass chip. They chose a directional coupler instead of a simple Y-splitter because the attenuation in the reference path was significantly reduced [17]. Yurtsever et al. integrated a multimode interference (MMI) splitter for OCT. They optimized the waveguide width to obtain low group velocity dispersion (GVD) [18]. Nguyen et al. fabricated two identical directional couplers for light splitting and balanced detection for swept-source OCT system-on-chip [19]. Akca et al. demonstrated a Mach-Zehnder interferometer (MZI)-based directional coupler with additional chromium electrical heaters placed on both the MZI arms to overcome fabrication-related performance degradation. They obtained a broad spectral flatness over 90 nm [20]. Another interferometry configuration with a non-uniform adiabatic coupler with weak wavelength dependence over 150 nm had been designed before. However, the interferometer length increased significantly to 3.5 mm [12].

In this paper, we designed a tandem MZDC configuration, obtained by connecting two MZDC through a short delay length. The delay length in the tandem MZDC is optimized to obtain the best response while maintaining the dedicated splitting ratio. To show how our configuration can improve OCT performance, we multiply the coupler output response with Gaussian windows and a single frequency test signal to observe the point spread function (PSF) response, which is similar to a signal reflection response in OCT. Results indicate that using tandem MZDC with more than 100 nm bandwidth compared with regular MZDC with around 40 nm bandwidth increases the sensitivity by more than 11 dB and signal-to-noise ratio by more than 24 dB.

## 2. Design

### 2.1. MZDC Parameter Design

**Figure 1:**
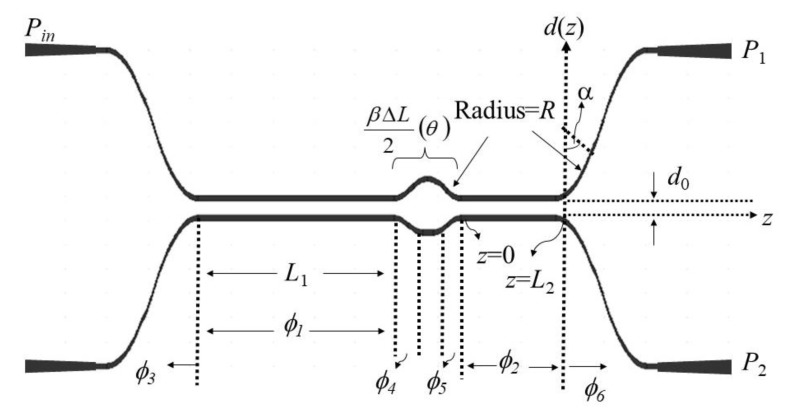
The schematic drawing for MZDC.

The MZDC with two DCs and one uncoupled region is shown in Figure 1.

**Figure 2:**
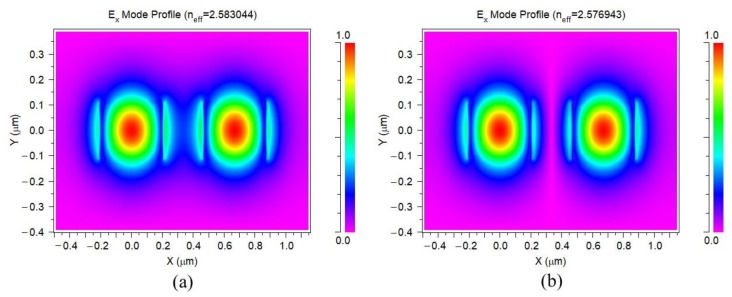
Effective indices simulation for (**a**) symmetrical and (**b**) antisymmetrical supermodes from two coupled straight waveguides.

The straight coupling length of the DC, *L*_c_, can be written in the following two types [15,16]:

(1) $$L_{c}=\frac{\lambda }{2\left(n_{s}-n_{a}\right)}$$

(2) $$L_{c}≅L_{0}e^{\frac{d}{D_{0}}}$$

where $n_{s}$ and $n_{a}$ are the effective refractive indices of the symmetric and antisymmetric supermodes in the straight waveguide, respectively, shown in Figure 2, through RSoft FemSIM simulation. *λ* is the operating wavelength, and *d* is the gap between two waveguides. *L*_0_ and *D*_0_ are phenomenological constants.

With the gap between two waveguides, *d* is equal to *d*_0_, *z* is the horizontal propagation length, the bending phase, $\phi_{\mathrm{bend}}$, on the MZDC can be written as

(3) $$\frac{\pi }{2L_{c}\left(d_{0}\right)}e^{\frac{-2R}{D_{0}}}\int_{0}^{R}e^{\frac{2\sqrt{R^{2}-z_{0}{}^{2}}}{D_{0}}}dz_{0}$$

where $z_{0}=z-L_{1}$ for the first DC,$\;z_{0}=z-L_{2}$ for the second DC, and *R* is the bend radius.

When a beam is transmitted from one input port with the power of *P*_in_, the output port with the *P*_1_ power can be used to express the splitting ratio *S* as *P*_1_/*P*_in_. *L*_1_ and *L*_2_ are representing $\phi_{1}$ and $\phi_{2}$, respectively, for the coupling phases from the straight coupler parts. $\phi_{I}$ and $\phi_{\mathrm{II}}$ are equal to the total phases from the first and second directional couplers of MZDC as $\phi_{1}+2\phi_{\mathrm{bend}}$ and $\phi_{2}+2\phi_{\mathrm{bend}}$, respectively.

(4) $$S=\frac{P_{1}}{P_{\mathrm{in}}}=cos^{2}\theta sin^{2}\left(\phi_{I}+\phi_{\mathrm{II}}\right)+sin^{2}\theta sin^{2}\left(\phi_{I}-\phi_{\mathrm{II}}\right)$$

(5) $$\theta =\frac{\beta \left(\lambda \right)\Delta L}{2}$$

(6) $$\phi_{1}=\int_{0}^{L_{1}}\frac{\pi }{2L_{c}}dz\;\mathrm{and}\;\phi_{2}=\int_{0}^{L_{2}}\frac{\pi }{2L_{c}}dz$$

where, *θ* is the phase difference of the uncoupled region, *β* is the waveguide propagation constant, Δ*L* is the length difference between the two arms of the Mach-Zehnder interferometer, and *z* is the traveling distance.

Based on the maximum flat wavelength response, the $\phi_{I}$ and $\phi_{\mathrm{II}}$, uncoupled region phase difference *θ*, and the splitting ratio *S* can be determined in the following [15]:

(7) $$\phi_{I}=\frac{3\pi }{8}\left(1+\frac{1}{N}\right)\;\mathrm{and}\;\phi_{\mathrm{II}}=\frac{3\pi }{8}\left(1-\frac{1}{N}\right)$$

(8) $$\theta =cos^{-1}\;\left(\sqrt{\left[\frac{sin\left(\frac{3\pi /2}{N}\right)}{-Nsin\left(3\pi /2\right)+sin\left(\frac{3\pi /2}{N}\right)}\;\right]}\right)$$

(9) $$S=\frac{sin\left(\frac{3\pi }{N}\right)}{4\left(N+sin\left(\frac{3\pi }{2N}\right)\right)}+sin^{2}\left(\frac{3\pi }{4N}\right)$$

**Figure 3:**
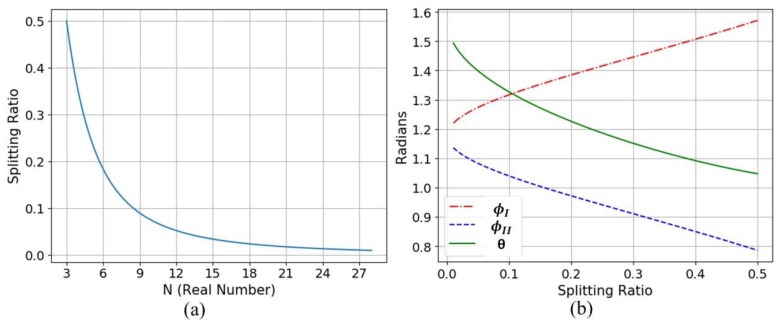
Relative curves of (**a**) splitting ratio and *N* (**b**) ratios with phases of $\phi_{I}$, $\phi_{\mathrm{II}}$ and *θ.*

The *N* values corresponding to the target splitting ratio can be obtained by using Equation (9) and shown in Figure 3a. Substituting the dedicated *N* value into Equations (7) and (8), we can find the total phase of the uncoupled region, *θ*, and coupled regions, $\phi_{I}$ and $\phi_{\mathrm{II}}$. The curves of $\phi_{I}$, $\phi_{\mathrm{II}}$, and *θ* versus the N related to splitting ration *N* are shown in Figure 3b. *N*, $\phi_{I}$, $\phi_{\mathrm{II}}$, and *θ* for different MZDC splitting ratios are listed in Table 1.

### 2.2. Schematic

**Figure 4:**
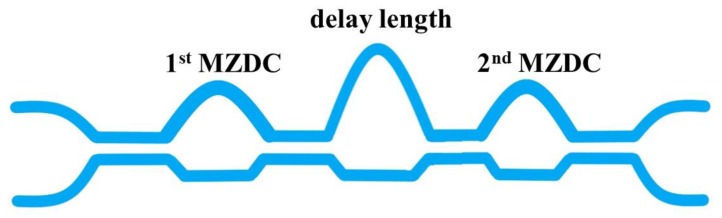
The schematic drawing for Tandem MZDC.

We added and adjusted a phase delay length between two MZDCs with different splitting ratios. The series of MZDC structure is called tandem MZDC and is shown in Figure 4. 

**Figure 5:**
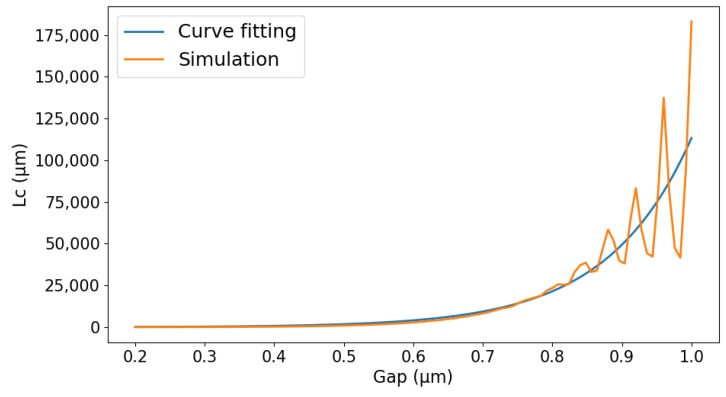
The relation between the DC gap and coupling length *L*_c._

In this paper, we simulated the waveguide in the transverse electric (TE) mode with 0.38 μm width, 0.22 μm height, 0.3 μm gap and 20 μm bend radius. In order to get *L*_0_ and *D*_0_ in Equation (2), the coupling length *L*_c_ and the gap of the directional coupler were simulated by RSoft FemSIM and shown in Figure 5. By using curve fittings, we found the value of *L*_0_ and *D*_0_ to be 28.212 and 0.121, respectively.

Since the phases from coupling regions of $\phi_{I}$ and $\phi_{\mathrm{II}}$ consist of straight and bend regions, the total phases can be expressed in respect of bend curve structure contribution and shown in the following:

(10) $$\phi_{I}=\frac{\pi }{2L_{c}}\left(L_{1}+2\times e^{\frac{-2R}{D_{0}}}\int_{0}^{R}e^{\frac{2\sqrt{R^{2}-z_{0}{}^{2}}}{D_{0}}}dz_{0}\right)=\frac{3\pi }{8}\left(1+\frac{1}{N}\right)$$

(11) $$\phi_{\mathrm{II}}=\frac{\pi }{2L_{c}}\left(L_{2}+2\times e^{\frac{-2R}{D_{0}}}\int_{0}^{R}e^{\frac{2\sqrt{R^{2}-z_{0}{}^{2}}}{D_{0}}}dz_{0}\right)=\frac{3\pi }{8}\left(1-\frac{1}{N}\right)$$

Under different splitting ratio conditions, the phases from coupled and uncoupled regions can be derived from Equations (7) and (8). Then using RSoft FemSIM to simulate $n_{s}$, $n_{a}$ and propagation constant β at different wavelengths, the straight length *L*_1_, *L*_2_, and Δ*L* of MZDC can be obtained by Equations (10), (11), and (5). All the parameters of *L*_1_, *L*_2_, Δ*L*, and mean percentage error under various splitting ratios are illustrated in Table 2. The straight lengths of *L*_1_ and *L*_2_ mentioned in this table are the phases for the total phases of $\phi_{I}$ and $\phi_{\mathrm{II}}$ subtracted from the 20-μm radius-based bend phase effects.

**Figure 6:**
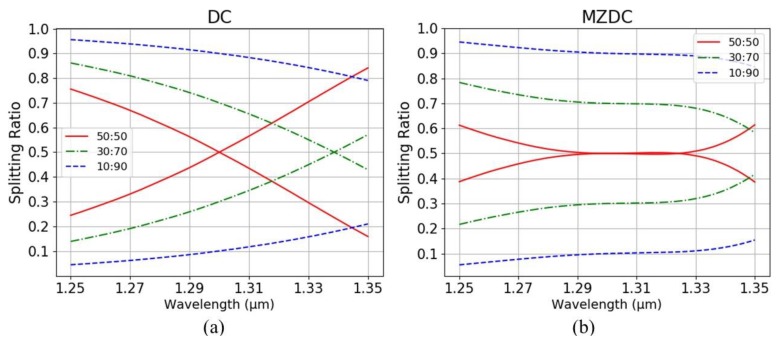
Splitting ratios of (**a**) DC and (**b**) MZDC.

To perform the arbitrary optical power dividers of MZDC, three splitting ratios of 0.1 (10:90), 0.3 (30:70), and 0.5 (50:50) are demonstrated. The wavelength response of MZDC splitting ratio is much flatter than DC. In Figure 6, the mean percentage error (MPE) of the coupler performance is calculated by averaging the percentage deviation from each wavelength actual response to the desired response. In the wavelength range of 1250 nm to 1350 nm, the MPE for directional coupler with 10:90 and 50:50 splitting ratio is 4.03% and 15.44%, respectively. Using the same parameters, the MPE of MZDC is 1.64% for 10:90 the splitting ratio and 2.99% for 50:50.

**Figure 7:**
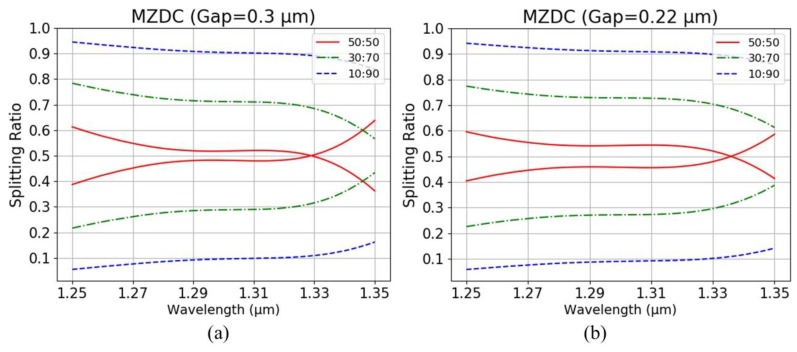
Excluding bend effect of MZDC with gap (**a**) 0.3 μm and (**b**) 0.22 μm.

When the bending effect on MZDC is not considered, $\phi_{I}$ and $\phi_{\mathrm{II}}$ are equal to $\phi_{1}$ and $\phi_{2}$, respectively. Then the MZDC splitting ratios of 0.1, 0.3, and 0.5 for 0.3 μm and 0.22 μm gaps are illustrated in Figure 7, which will deviate from the ratio values and wavelength flatness. It also shows that a smaller gap between the coupler causes a higher bend effect and will cause more deviation. We compare the average percentage error of MZDC splitting ratios with two gaps, 0.3 and 0.22 μm, and the including/excluding bend effects are also listed in Table 3.

The MZDC flatness parameters are taken to design tandem MZDC with the same coupling power, which means that the first DC ratio with $\phi_{I}$ phase of MZDC is the same as the first MZDC and second DC with $\phi_{\mathrm{II}}$ as the second MZDC. When the 0.1 splitting ratio is considered, the coupling phase $\phi_{I}$ from the first DC is taken as 1.317 rad and $\phi_{\mathrm{II}}$ from the second DC as 1.039 rad, shown in Figure 3b. The phase difference of MZDC uncoupled region, *θ*, is taken to optimize the flat wavelength response using the DC constant phase performance. Then MZDC power splitter with insensitive wavelength response shows a non-linear output phase. Therefore, the delay length, dL, of tandem MZDC will be varied to get the flattest response in the wide wavelength range while maintaining the dedicated coupling power ratio.

**Figure 8:**
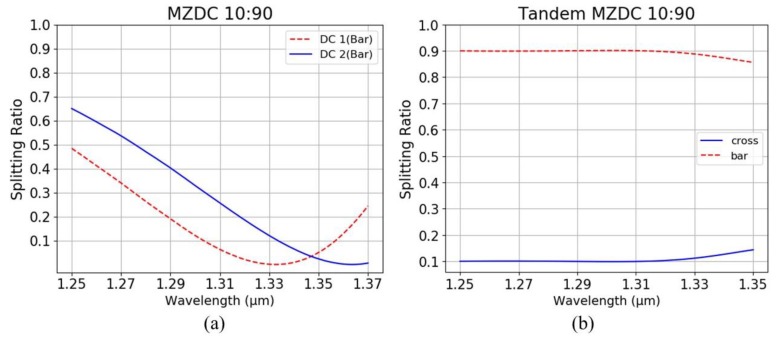
(**a**) Two DC splitting ratios from the MZDC structure, and (**b**) MZDC 06:94+ dL (0.52 μm) + MZDC 25:75 from the tandem MZDC structure.

In Figure 8, the ratios of the central wavelength, 1.31 μm, of MZDC are 6:94 and 25:75 for the first DC, DC 1, and the second DC, DC 2, ratios, respectively. The first and second MZDC from tandem MZDC will take these same ratios to optimize the delay length as 0.52 μm for the flattest wavelength response. Hence, using two wavelength-insensitive MZDCs as the coupling functions in a tandem MZDC could demonstrate a more extensive decoupled phase term to maximize the flat wavelength response.

### 2.3. OCT Brief Theory

OCT is an imaging modality with resolution in the µm range and depth in the mm range. Spectral domain-OCT is based on the principle of low coherence interferometry while swept source -OCT uses a wavelength-swept laser. Both OCT technologies need further intensive steps in signal processing [1]. The image resolution is one of the most important parameters governing OCT image quality. In contrast to standard microscopy, OCT can achieve better axial resolution independent of the beam focusing and spot size. The bandwidth of the light source determines the image resolution. For a Gaussian-shaped spectrum, the axial resolution is:

(12) $$\Delta z=\frac{2ln2\lambda^{2}}{\pi \Delta \lambda }$$

where, Δ*z* is the full-width-at-half-maximum (FWHM) of the autocorrelation function, Δ*λ* is the optical power spectrum FWHM, and λ is the central wavelength of tof the autocorrelation functionhe light source. A Gaussian-shaped light source spectrum is convenient to be used in modeling OCT because it approximates the shape of actual light sources and also has Fourier transform properties. The normalized Gaussian function *S(k)* and its inverse Fourier transform *γ(z)* are given in the following: [1]

(13) $$\gamma \left(z\right)=e^{-{\left(z\right)}^{2}\Delta^{2}}\to S\left(k\right)=\;\frac{1}{\Delta k\sqrt{\pi }}e^{-{\left(\frac{k-k_{0}}{\Delta k}\right)}^{2}}$$

Here, *k_0_* represents the central wavenumber of the light source spectrum, and Δ*k* represents its spectral bandwidth, corresponding to the half-width of the spectrum at 1/*e* of its maximum. The inverse Fourier transform γ(z) is the axial point spread function (PSF) in OCT imaging systems.

Spatial response intensity from a reflection in OCT can be calculated in the following: [1]

(14) $$I_{D}\left(z\right)=(\gamma \left(z\right)⊗\overset{N}{\underset{n=1}{\sum }}\sqrt{R_{R}R_{S}}\left(\delta \left[\left(z\pm 2\left(z_{R}-z_{S}\right)\right)\right]\right)$$

where, γ(z) is a gaussian function in the spatial domain with FWHM equal to a light source coherence length, $\sqrt{R_{R}R_{S}}$ is a coefficient of reflection, and the $\delta \left[\left(z\pm 2\left(z_{R}-z_{S}\right)\right)\right]$ is a result of Fourier transform from a single reflection. Therefore, the convolution between a delta function and Gaussian function could demonstrate the reflective signal. This equation is usually called the cross-correlation term, which is the desired component in the OCT signal. 

To analyze the performance of MZDC and Tandem MZDC, we multiplied each output with a Gaussian spectrum and a single frequency signal. The Gaussian spectrum signal has a 1310-nm central wavelength and 100-nm bandwidth, which equals the 7.5-µm axial resolution. Fourier transform is then applied on the signal to obtain a spatial PSF response. We compared the PSF response from the MZDC and the tandem MZDC concerning their sensitivity and signal-to-noise ratio (SNR).

## 3. Experiments and Results

**Figure 9:**
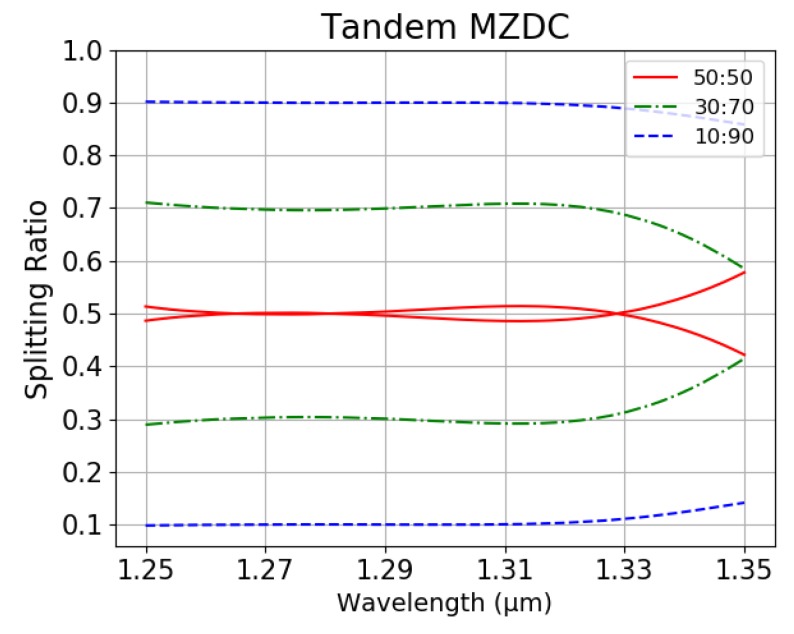
Splitting ratio of Tandem MZDC.

We optimized the delay length (dL) parameter in tandem MZDC, shown in Figure 4, with first MZDC and second MZDC parameters obtained by the MZDC design. Unlike DC, MZDC with the flat wavelength response has a non-linear output phase. Table 4 shows parameters, mean percentage error, and standard deviation for tandem MZDC with different splitting ratios. For all of the designed tandem MZDC, we obtained less than 0.027 standard deviations and less than 2% mean percentage error. Compared with 40 nm flat-response MZDC, the tandem MZDC output spectrum demonstrates a flatter response for more than 100 nm, as shown in Figure 9. 

**Figure 10:**
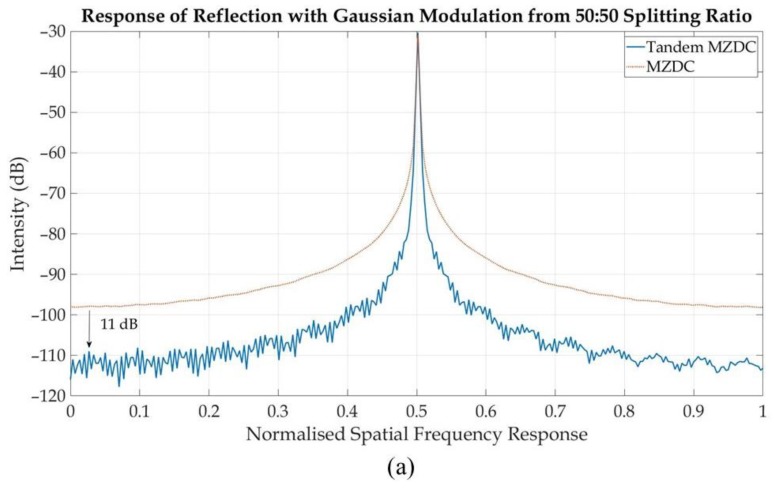

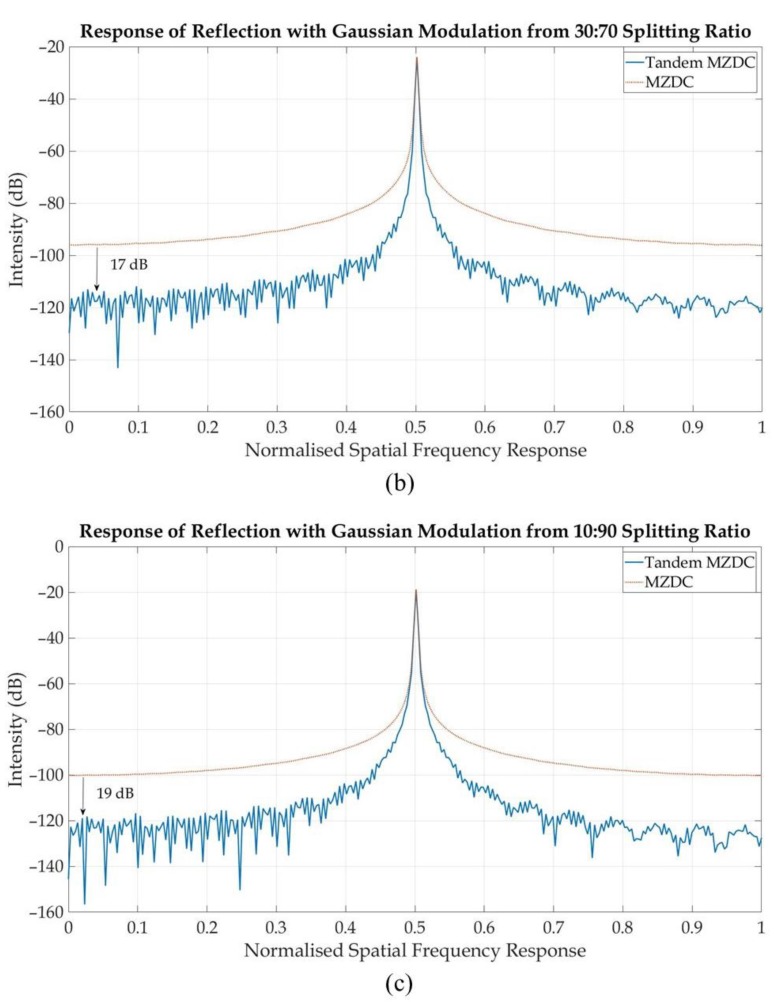
Spatial response of MZDC and tandem MZDC with: (**a**) 50:50 splitting ratio, (**b**) 30:70 splitting ratio, and (**c**) 10:90 splitting ratio.

All of these results from tandem MZDC and regular MZDC with the same splitting ratio are then multiplied with a gaussian function and single-frequency signal. The SNR for fast Fourier transform (FFT) plot spectrum will ideally be an impulse of the signal in dB compared with all (integral) of the background noise. For our case, because our signal is a cosine modulated with gaussian function, the FFT result will not be a single peak but widening impulse, hence the SNR is calculated by comparing the ratio of this widening impulse with all of the noise floor. The Fourier transform from these signals is shown in Figure 10. We can conclude that the SNR of tandem MZDC is better than MZDC.

**Figure 11:**
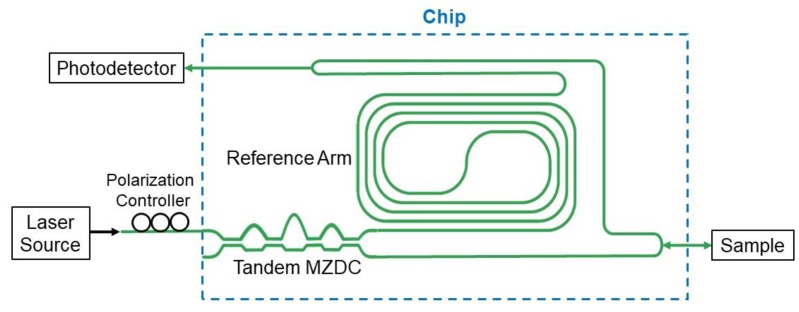
Optical coupler-based OCT interferometer.

The swept source-OCT interferometry configuration could include the laser source, optical power coupler, reference arm, and photodetector, as shown in Figure 11. The main goal for this paper is theoretically to demonstrate 24-dB signal-to-noise ratio improvement through the broadband coupler, Tandem MZDC.

## 4. Discussion 

In designing a broad bandwidth response coupler, we are tuning the value of dL, and the best parameter for the optical delay length in tandem MZDC is always located about 0.5–1.5 µm. Therefore, the fabrication process precision is crucial to the wavelength insensitivity response.

In Table 4, tandem MZDC with a 10:90 splitting ratio has the lowest mean percentage error, hence 10:90 tandem MZDC should own the best SNR, as shown in Figure 9. We can conclude that the small MPE and flat wavelength response can be utilized to get better SNR. In Table 5, we compared the SNRs of MZDC and tandem MZDC with different splitting ratios. After tandem MZDC replaced the MZDC as the OCT coupler, we observed at least 24-dB SNR improvement.

## 5. Conclusions

We have simulated broadband tandem MZDC with a TE mode. A broadband coupler with an arbitrary splitting ratio can be optimally designed through tandem MZDC, in which the bandwidth demonstrates more than 100 nm. By comparing the performance with regular MZDC, tandem MZDC has better sensitivity and better SNR. Hence, our design has potential for OCT images on-chip applications.

## Figures and Tables

**Table 1:** Splitting ratio parameters with *N*, $\phi_{I}$, $\phi_{\mathrm{II}}$_,_ and *θ.*

Splitting Ratio	N	$\phi_{I}\;(\mathrm{rad})$	$\phi_{\mathrm{II}}\;(\mathrm{rad})$	*θ* (rad)
10:90	8.485	1.317	1.039	1.327
30:70	4.402	1.446	0.910	1.15
50:50	3	1.571	0.785	1.047

**Table 2:** MZDC design parameters.

Splitting Ratio	10:90	30:70	50:50
*L*_1_ (μm)	93.77	103.22	112.39
*L*_2_ (μm)	73.40	63.95	54.77
ΔL (μm)	0.220	0.191	0.174
Mean percentage error	1.64%	2.87%	2.99%

**Table 3:** Mean percentage error of MZDC including/excluding bend effects.

Splitting Ratio		10:90	30:70	50:50
Including bend part mean percentage error	Gap = 0.3 μm	1.64%	2.87%	2.99%
	Gap = 0.22 μm	1.29%	1.98%	1.93%
Excluding bend part mean percentage error	Gap = 0.3 μm	1.75%	3.37%	4.01%
	Gap = 0.22 μm	1.67%	3.48%	4.57%

**Table 4:** Parameters design of Tandem MZDC.

Splitting Ratio	10:90	30:70	50:50
First MZDC	06:94	02:98	0:100
Second MZDC	25:75	63:37	50:50
dL (μm)	0.52	0.55	1.49
Mean percentage error	0.61%	1.48%	1.19%
Standard Deviation	0.020	0.027	0.011

**Table 5:** SNR from spatial response of MZDC and tandem MZDC.

Splitting Ratio	Tandem MZDC SNR (dB)	MZDC SNR (dB)
10:90	99.42	65.45
30:70	84.11	58.94
50:50	87.82	53.98
